# Vitamin D Supplement Use Is Associated with Better Status, Yet Most Biochemical Vitamin D, Iron and Vitamin B12 Deficiency Is Unrecognised and Unsupplemented: A Cross-Sectional Study of a Screened Corporate Workforce in Poland

**DOI:** 10.3390/nu18213611

**Published:** 2026-10-30

**Authors:** Dominik Swadźba, Sebastian Makuch, Siddarth Agrawal

**Affiliations:** 1 Faculty of Medicine, Andrzej Frycz Modrzewski Krakow University, 30-705 Kraków, Poland; 2 Housemed S.A., 30-703 Kraków, Poland; 3 Department of Clinical and Experimental Pathology, Wrocław Medical University, 50-368 Wrocław, Poland; s.makuch@umw.edu.pl; 4 Labplus R&D, 50-266 Wrocław, Poland

**Keywords:** vitamin D, iron deficiency, ferritin, vitamin B12, dietary supplements, micronutrient status, screening, occupational health, Poland

## Abstract

Background/Objectives: Whether self-directed supplement use reaches people who are deficient is unclear. We examined vitamin D, iron and vitamin B12 status and supplement use in a screened workforce. Methods: Cross-sectional analysis of 793 employees (75.2% women, aged 18 to 67) in Poland sampled from April to June 2026; 25-hydroxyvitamin D [25(OH)D], ferritin and vitamin B12 were measured by automated immunoassay, and supplement use was ascertained from an open inventory. Deficiency was defined as 25(OH)D < 30 ng/mL, ferritin < 30 µg/L and vitamin B12 < 200 pg/mL. Results: Deficiency was common (vitamin D 53.2%; iron 29.4%, 38.3% of women; vitamin B12 3.0%). Vitamin D users reached the target more often than non-users (77.4% versus 38.5%; adjusted odds ratio 5.5, 95% CI 3.7 to 8.3; 1.8 in a sensitivity scenario that reclassified prompted reports as use); with 22 iron and 17 vitamin B12 users, no association could be characterised. Few deficient participants listed the corresponding supplement (vitamin D 9.0%, iron 2.2%, vitamin B12 0%; 32.1% and 9.1% with a prompted item). Among deficient respondents, 70.2%, 69.2% and 86.4% (vitamin D, iron, vitamin B12) reported neither a prior diagnosis nor a supplement, and routine characteristics discriminated deficiency poorly (*C*-statistic 0.58 for vitamin D, 0.57 for iron within women). Conclusions: Vitamin D supplement use was associated with better status, but most biochemical deficiency occurred in people who reported neither a diagnosis nor a supplement and could not be identified from routine characteristics.

## 1. Introduction

Deficiencies of vitamin D, iron and vitamin B12 remain common in adults of working age. Vitamin D insufficiency affects a substantial proportion of European adults, with the exact figure depending on the threshold applied [1,2,3], and surveys in Poland have reported 25(OH)D below 30 ng/mL in about 90% of adults sampled in winter and spring [4,5]. Iron deficiency affects roughly 9 to 11% of reproductive-age women and frequently precedes anaemia [6,7], with depleted or small iron stores (ferritin at or below 30 µg/L) in 40 to 55% of European women of reproductive age [8], and vitamin B12 deficiency affects approximately 6% of adults younger than 60 years [9,10,11]. These conditions are consequential before they become overt: iron deficiency without anaemia is associated with fatigue and reduced physical capacity and improves with repletion [12,13], and vitamin D and B12 deficiencies carry musculoskeletal and neurological consequences [14,15].

Adults increasingly respond to this burden on their own initiative. Roughly half of adults in high-income countries report taking at least one dietary supplement; vitamin D is among the most frequently used products, and use has risen steadily for two decades [16,17]. Most users start supplements to “improve overall health” rather than on medical advice [18]. Supplementation therefore functions as a large, self-directed public-health intervention.

Two questions follow, and they are distinct. The first is whether unsupervised supplement use is accompanied by adequate biochemical status. Randomised trials establish dose–response relationships under monitored conditions [19,20,21], but real-world attainment is less well described; one clinic-based analysis found that vitamin D adequacy improved only slowly despite intensive testing and widespread supplement use [22]. The second question, less often asked, is whether supplementation reaches the people who are deficient. These can diverge sharply: if supplement use is driven by health-consciousness rather than by measured status, a population may combine high supplement uptake with a large, unreached deficiency burden.

Answering the second question requires knowing both biochemical status and supplement use in the same unselected individuals, with supplement use ascertained independently of the laboratory result. Workplace screening programmes offer that opportunity, reaching working-age adults who have limited preventive-care contact and in whom general health checks have otherwise shown limited benefit [23,24].

Using such a programme, we asked: (i) how common are vitamin D, iron and vitamin B12 deficiency in an ostensibly healthy workforce; (ii) is supplement use associated with better biochemical status, and what proportion of users reach guideline targets; (iii) what proportion of the deficient population reports taking the corresponding supplement, and how does the remaining burden divide between people who report a prior diagnosis and those who do not; and (iv) can deficiency be identified from routinely available characteristics, which determines whether supplementation could be targeted without testing. Because the design is cross-sectional, the study describes associations; it cannot establish that supplementation caused the better status observed in users.

## 2. Materials and Methods

### 2.1. Study Design and Population

This was a cross-sectional analysis of de-identified records from a workplace preventive-health programme delivered to employees of a large corporate employer in Poland with predominantly office-based work. The programme was offered to 1500 employees; 793 (52.9%) took part, and all 793 were included, without exclusion criteria. Blood was drawn between 21 April and 16 June 2026; the 25(OH)D result was reported in April for 429, in May for 336 and in June for 26 of the 791 participants with a 25(OH)D result, in every case within 4 days of the first result from the same sample, and two participants had a blood count and glycated haemoglobin only. Participants underwent venous laboratory testing and completed a structured medical interview administered by a certified clinical decision support system (LabTest Checker, Labplus S.A., Wrocław, Poland; a CE-marked class IIa medical device under Regulation (EU) 2017/745). Participation was voluntary, so the sample is self-selected in both directions: health-conscious employees and employees who suspected a problem may both have been more likely to attend. The sex and age structure of the invited workforce was not available to the investigators. Reporting follows the STROBE recommendations for cross-sectional studies [25] (checklist in Table S6).

The laboratory panel was partly individualised: beyond a common core panel, additional analytes were added according to participant choice. The number tested therefore varied by analyte, and denominators are reported for each measurement.

### 2.2. Laboratory Measurements and Definitions of Deficiency

All samples were analysed by Diagnostyka S.A. (Kraków, Poland), a nationwide network of medical laboratories whose central laboratories hold PN-EN ISO 15189 [26] accreditation (certificates AM 003, AM 006, AM 009 and AM 014) and ISO 9001 [27] certification [28]. Polish law requires medical laboratories to carry out continuous internal quality control and to ensure external quality control [29,30]. For the analytes studied, laboratories in the network take part, according to the laboratory, in the following external quality assessment schemes: for 25(OH)D, the Randox RIQAS immunoassay or immunoassay speciality scheme (Randox Laboratories, Crumlin, UK) or the Labquality scheme for vitamins A and E and vitamin D metabolites (scheme 2480; Labquality, Helsinki, Finland), as no scheme of the Central Centre for Quality Assessment in Laboratory Diagnostics covers this analyte; for ferritin and vitamin B12, the Central Centre’s extended immunochemistry scheme together with the RIQAS monthly immunoassay or specific proteins scheme or the Labquality Hormones A scheme (2300); for blood counts, the Central Centre’s haematology scheme and the RIQAS Haematology or Labquality blood count scheme (4100). The laboratory confirmed the equipment used across its network for the analytes studied: 25(OH)D by chemiluminescence immunoassay on DiaSorin LIAISON XL (DiaSorin, Saluggia, Italy) and Abbott Alinity i (Abbott Laboratories, Abbott Park, IL, USA) analysers; ferritin by spectrophotometric immunoturbidimetric assay on clinical chemistry analysers (Roche cobas c series and Integra 400, Roche Diagnostics, Mannheim, Germany; Abbott Alinity c); vitamin B12 by electrochemiluminescence or chemiluminescence immunoassay (Roche cobas e series, Abbott Alinity i, DiaSorin LIAISON XL); and blood counts by fluorescence flow cytometry (Sysmex XN series, Sysmex, Kobe, Japan; Mindray BC-6000 series, Mindray, Shenzhen, China). The allocation of individual samples to analysers was not recorded. The assay-specific reference intervals on the vitamin B12 reports indicate that 84% of samples (663/791) had vitamin B12 measured on a Roche analyser, 6% (44/791) on an Abbott analyser and 11% (84/791) on a third platform (Table S3), whereas the 25(OH)D and ferritin reports carried uniform laboratory ranges that do not identify the analyser, so the split of 25(OH)D measurements between the two chemiluminescence assays is unknown.

Both 25(OH)D assays appear on the list of assays certified by the CDC Vitamin D Standardization-Certification Program, whose criteria are a mean bias within 5% of the reference measurement procedure and imprecision below 10% [31]. The LIAISON 25 OH Vitamin D TOTAL assay has a measuring range of 4 to 150 ng/mL, equimolar cross-reactivity for 25(OH)D2 and 25(OH)D3, 1.3% cross-reactivity for 3-epi-25(OH)D3, and manufacturer-reported total coefficients of variation of 10.4% at 20 ng/mL, 10.6% at 24 ng/mL and 10.3% at 57 ng/mL [32]. The Abbott 25-OH Vitamin D assay is documented for its ARCHITECT version as traceable to the isotope-dilution liquid chromatography–tandem mass spectrometry reference measurement procedure and NIST SRM 2972, with a reportable range of 3.4 to 155.9 ng/mL, cross-reactivity of 81 to 82% for 25(OH)D2, 0.5 to 1.3% for 3-epi-25(OH)D3 and 102 to 189% for 24,25-dihydroxyvitamin D3, and total coefficients of variation of 3.2% at 21 ng/mL, 3.1% at 31 ng/mL and 3.1% at 72 ng/mL [33]; performance data specific to the Alinity i version and the laboratory’s own internal quality control data for the sampling period were not available to the authors. The two assays therefore differ in imprecision near the 30 ng/mL threshold and in their response to 24,25-dihydroxyvitamin D3, which is one reason both thresholds are reported. All results were within the measuring ranges of the assays; the highest was 154.2 ng/mL. On the Roche platform, the vitamin B12 reference interval corresponds to the Elecsys Vitamin B12 II assay. Serum vitamin B12 and ferritin assays are not harmonised across manufacturers, and a single threshold was applied to all platforms. The 25(OH)D is reported in ng/mL (multiply by 2.496 for nmol/L), ferritin in µg/L and vitamin B12 in pg/mL (multiply by 0.738 for pmol/L).

Deficiency was defined using international guideline thresholds rather than laboratory reference limits. For vitamin D, the primary threshold was 25(OH)D < 30 ng/mL, the target of the Endocrine Society 2011 and Polish 2023 guidelines [14,34], with <20 ng/mL reported alongside it in every analysis because the 30 ng/mL threshold is debated for asymptomatic adults, and the 2024 Endocrine Society guideline no longer defines a target for them [35]; the Endocrine Society terms 20 to 29.9 ng/mL insufficiency and below 20 ng/mL deficiency [14], and for brevity, “deficient” is used here for any value below the primary threshold. For iron, the primary threshold was serum ferritin < 30 µg/L, the threshold adopted by clinical guidelines across indications [36] and corresponding to absent stainable bone-marrow iron; the World Health Organization threshold for apparently healthy adults, <15 µg/L [37], was reported as a sensitivity analysis. For vitamin B12, the threshold was serum vitamin B12 < 200 pg/mL [9], which is close to the lower reference limit of the Roche assay (197 pg/mL). Throughout, “deficiency” denotes a concentration below the stated threshold on a single measurement; no clinical signs, symptoms or functional markers (methylmalonic acid, homocysteine, holotranscobalamin) entered the definitions, and inflammation markers were part of the optional panel: C-reactive protein was measured in 23 participants (median 0.9 mg/L, range 0.3 to 10.6; 3 above 5 mg/L; 3 with ferritin below 30 µg/L) and transferrin saturation in 1. Ferritin rises with inflammation, so a value below 30 µg/L is specific for depleted stores, whereas inflammation can raise ferritin above 30 µg/L and mask deficiency. The interview items on infection within two weeks, dental disease and flare of inflammatory disease that the decision support system uses to qualify a ferritin result are presented after a ferritin result at or above 30 µg/L (the dental and flare items to none of the 232 participants with ferritin below 30 µg/L, the infection item to 47 of them), so they cannot estimate masked deficiency. Among the 557 participants with ferritin at or above 30 µg/L, the infection item was presented to 551, the dental item to 441 and the flare item to 361; of the 285 with ferritin of 30 to 69 µg/L, 284 were presented at least one of the three items, and 98 answered yes to at least one. Two scenarios indicate how much masked deficiency could change the prevalence: counting those 98 as iron-deficient under the WHO threshold for inflammation (<70 µg/L) gives (232 + 98)/789 = 41.8%, and counting all 285 participants with ferritin of 30 to 69 µg/L gives (232 + 285)/789 = 65.5%. Neither is a bound on the true prevalence because the conditions were self-reported, two of the three items were presented to a subset, and no inflammation marker was measured in most participants. Anaemia was defined by World Health Organization criteria (haemoglobin < 12 g/dL in women, <13 g/dL in men) [38] and is reported for context.

### 2.3. Ascertainment of Supplement Use and Prior Diagnosis

Supplement use was ascertained with two instruments. The primary instrument was a single open-ended item administered to all participants near the start of the interview, which asked what medicines and supplements they currently take, explicitly including prescription drugs, over-the-counter products, supplements and vitamins; entries are product names from the system’s product dictionary. The item was presented to all 793 participants and was not conditioned on any laboratory result by the system; 383 participants (48.3%) entered at least one product. The item asked about current use at the time of the interview and made no reference to the blood draw. The interview was completed after the laboratory had reported the results (on the day of the first result in 401 participants; median 0 days, 75th percentile 5, maximum 25) because the result-triggered items require them, so participants may have had access to their results before completing the inventory, and a participant who started a supplement after seeing a low result within that interval would be classified as a user, while the measured concentration reflects status before supplementation. The share of participants reporting use did not differ between those interviewed on the day of the result and later (86/401, 21.4%, versus 82/390, 21.0%), and the comparison of users with non-users was repeated within these two groups (Section 2.5). The 1038 entries from 383 participants were mapped to nutrient categories by explicit rules written out for the revised analysis and applied to every entry (Table S1): a product was attributed to a nutrient if the nutrient was named in the product name (for example “witamina D3”, “żelazo”, “B12”) or if the product was a single-nutrient branded preparation of that nutrient (for example Vigantol, Devikap, Ibuvit D, Sorbifer Durules, Tardyferon, DiaB12). Attribution was by name; product composition was not consulted. Combination products in which the nutrient was named were counted regardless of dose: 25 vitamin D users reported vitamin D3 with vitamin K2, 2 reported calcium with vitamin D3, 5 reported a vitamin A, D, E and K product, and 10 iron users reported iron combined with folic acid or vitamin C. Multivitamin and multimineral products that did not name the nutrient (9 entries from 8 participants) and B-complex products that did not name vitamin B12 (22 participants) were not attributed in the primary analysis and were examined in sensitivity analyses. Two participants listed two vitamin D products and one listed two iron products, so product counts in Table S1 exceed user counts by that number. Dose was not a structured field; 47 of the 170 vitamin D entries carried a dose in free text (1000 IU in 3, 2000 IU in 34, 4000 IU in 10) and were not analysed further. Formulation, frequency, duration and adherence were not recorded.

The second instrument comprised condition-specific prompted questions on current supplement use (“Do you take vitamin D or combination products containing vitamin D?”; “Do you take iron or vitamins with iron?”; “Do you take vitamin B12, orally or by injection?”) that the interview presents only on a trigger. The vitamin D item is triggered by a 25(OH)D result below 30 or above 50 ng/mL; it was presented to 378 of the 421 participants below 30 ng/mL (10 of them left it unanswered) and to 61 of the 90 above 50 ng/mL. Of the 43 participants below 30 ng/mL to whom it was not presented, 25 had already listed a vitamin D product in the open inventory, consistent with the item being withheld when the product dictionary records vitamin D, and 18 had not, for reasons that could not be reconstructed from the export. The iron item is triggered by low ferritin or by anaemia; it was presented to 209 of the 232 participants with ferritin below 30 µg/L (3 unanswered) and to 33 with ferritin at or above 30 µg/L. The vitamin B12 item is not linked to the vitamin B12 result; it was presented to 52 participants, 3 of them with vitamin B12 below 200 pg/mL (Table S5). These were not used to define supplement status in the primary analysis because their ascertainment is conditioned on the laboratory value and would bias any comparison of status by supplement use. Because a prompted question captures use that an open inventory omits, we report, as an analysis added during peer review, the proportion of deficient participants who reported the supplement on either instrument, and a sensitivity analysis in which prompted reports are reclassified as use (Section 2.5).

Prior recognition of deficiency was taken from condition-specific interview items asking whether a clinician had previously diagnosed the corresponding condition: one item for vitamin D deficiency; four items for iron (iron deficiency, iron-deficiency anaemia, anaemia, and current medical care for anaemia); and two items for vitamin B12 (vitamin B12 deficiency and vitamin B12-deficiency anaemia). The items are presented on an abnormal result, and the export records every presented item, answered or not. The vitamin D item was presented to all 421 participants below 30 ng/mL and answered by 356 (84.6%); at least one iron item was presented to 231 of the 232 with ferritin below 30 µg/L (the iron-deficiency item to 223) and answered by 211 (90.9%); and at least one vitamin B12 item was presented to all 24 with low vitamin B12 and answered by 22 (91.7%) (Table S5). A participant was classified as reporting a prior diagnosis if any presented item was answered yes, as reporting none if every answered item was answered no, and as a non-respondent if no presented item was answered. Non-response was retained as a separate category in the partition of the deficient population. The share of deficient participants who reported neither a prior diagnosis nor a supplement was estimated among respondents to the prior-diagnosis items (the primary estimate; supplementing participants count as respondents only if they answered the item) and, as an alternative, among all deficient participants with non-respondents counted as undiagnosed.

### 2.4. Covariates

Age, sex and current smoking (cigarettes, e-cigarettes or other tobacco products; missing for 6 participants) were recorded for all participants. BMI was calculated from reported height and weight and was unavailable for 22 participants who did not report height (6) or weight (19); reported values (height 150 to 195 cm, weight 43 to 177 kg) were all plausible, and none was excluded. Pregnancy was asked of 568 of the 596 women, and none reported being pregnant. Month of sampling was taken from the date of the 25(OH)D result. Regular physical activity (at least 150 min per week of moderate or 75 min of vigorous activity) was asked of 277 participants (273 answered) through interview branching whose trigger could not be reconstructed from the export, so the comparison of users and non-users on this item is descriptive and conditional. Income, education and occupational grade were not recorded.

### 2.5. Statistical Analysis

Concentrations are summarised as median (interquartile range, IQR) and compared between supplement users and non-users using the Mann–Whitney U test; attainment of the target is compared with Fisher’s exact test. Proportions are reported with Wilson 95% confidence intervals (CI). The association between supplement use and attainment of the biochemical target was estimated as a crude odds ratio and by logistic regression adjusted for age, sex, BMI, current smoking and month of sampling (three categories), and the difference in 25(OH)D by linear regression with the same covariates on the raw scale, with a log-scale model as a check; comorbidities (median 0) were not included. Two sensitivity scenarios examine how under-ascertainment of vitamin D use by the open inventory could affect the comparison of users with non-users; they are scenarios under stated assumptions, not bounds on the true association, because they address under-ascertainment only and leave confounding and reverse causation untouched. Scenario A assumes that every report on the prompted item is true current use, reclassifies those participants as users, and assumes that no user was missed among the 280 participants with 25(OH)D of 30 to 49.9 ng/mL, who were never prompted; because reclassification adds users only below 30 and above 50 ng/mL, this scenario moves the estimate towards the null as far as under-ascertainment plausibly can. Scenario B additionally assumes that the open inventory missed users in the 30 to 49.9 ng/mL band at the same rate as above 50 ng/mL, where 42 of 84 users were captured; the implied 88 additional users were drawn at random from non-users in the band in 200 replicates, and the median adjusted odds ratio is reported. A third analysis addresses the timing of the interview: the comparison was repeated among participants interviewed on the day the result was reported and among those interviewed later. The deficient population was partitioned into mutually exclusive groups: taking the corresponding supplement but still below target; not taking it and reporting a previous diagnosis; not taking it and reporting no previous diagnosis; and not taking it with the prior-diagnosis item unanswered. Discrimination of deficiency by age, sex, BMI and smoking was assessed by multivariable logistic regression and summarised with the *C*-statistic, with optimism estimated by Harrell’s bootstrap: in each of 1000 resamples (seed 7), the model with the fixed covariate set was refitted, the difference between its *C*-statistic in the resample and in the original data was recorded, and the mean difference was subtracted from the apparent *C*-statistic; no variable selection was performed, and calibration was not assessed. For iron deficiency, the model was also fitted within women. Because only one man had ferritin below 15 µg/L, the model at that threshold was estimated by Firth’s penalised likelihood with profile-likelihood intervals. Sensitivity analyses at the alternative thresholds (25(OH)D < 20 ng/mL; ferritin < 15 µg/L) were specified before the first analysis. The remaining sensitivity analyses were not prespecified: counting B-complex products as vitamin B12 exposure and multivitamin products as vitamin D and iron exposure, the prompted-item analyses and the reclassification analyses were added after the first analysis, in response to peer review, and the respondent-based estimate of the partition, adopted as the primary estimate, was introduced at the same stage. Analyses were descriptive and estimation-focused; confidence intervals were not adjusted for multiple comparisons. Analyses were performed in Python 3.13 (Python Software Foundation, Beaverton, OR, USA) with pandas 3.0, SciPy 1.18, statsmodels 0.15 and scikit-learn 1.9.

### 2.6. Ethics

The study was a retrospective analysis of de-identified programme records. The study was conducted in accordance with the Declaration of Helsinki and approved by the Ethics Committee of Andrzej Frycz Modrzewski Krakow University (opinion no. KB/UAFM/50/2026, 16 July 2026), after the programme had ended and before the analysis began. Participants had provided written informed consent at enrolment for participation in the programme and for the scientific analysis of their anonymised data.

## 3. Results

### 3.1. Participant Characteristics

The 793 participants had a mean age of 39.4 years (standard deviation, SD 9.3; range 18 to 67) and 596 (75.2%) were women, a sex imbalance that shapes the iron findings reported in Section 3.2. Only 39 participants were younger than 25, and three were 65 or older. The mean BMI was 25.3 kg/m^2^ (SD 4.8), and 127 of 787 participants with valid smoking data (16.1%) were current smokers. Most reported no previously diagnosed chronic disease (median 0, IQR 0 to 1). Among the 791 participants with a 25(OH)D result, vitamin D supplement users and non-users were alike in age (39.3 versus 39.4 years), sex (126/168, 75.0%, versus 468/623, 75.1%, women) and BMI (25.3 versus 25.3 kg/m^2^); users smoked less often (13.2% versus 17.0%) and, among the 273 participants who answered the physical-activity item, more often reported regular activity (45/60, 75.0%, versus 110/213, 51.6%). Users were sampled earlier in the programme (107/168, 63.7%, in April versus 322/623, 51.7%; *p* = 0.007). Characteristics are shown in Table 1.

### 3.2. Deficiency Prevalence and Supplement Use

A 25(OH)D below 30 ng/mL was the most common finding, affecting 421 of the 791 participants tested (53.2%); 187 (23.6%) were below 20 ng/mL (139/594 women, 23.4%; 48/197 men, 24.4%), and prevalence at 30 ng/mL did not differ by sex (312/594 women, 52.5%; 109/197 men, 55.3%). Iron deficiency affected 232 of 789 (29.4%), almost entirely women (227/592, 38.3%, versus 5/197 men, 2.5%); 92 participants (11.7%) had ferritin below 15 µg/L, 91 of them women. Anaemia accompanied iron deficiency in 28 of 232 (12.1%) at the 30 µg/L threshold (27 of 592 women, 4.6%, and 1 of 197 men) and in 23 of 92 (25.0%) at 15 µg/L, and 19 of 232 had a mean corpuscular volume below 80 fL. Low serum vitamin B12 (24/791, 3.0%; median 175 pg/mL, range 110 to 199) and anaemia (30/792, 3.8%) were uncommon; of the 24, one had a mean corpuscular volume above 98 fL and two were anaemic, and a further 118 participants (14.9%) had vitamin B12 between 200 and 300 pg/mL, a range in which functional deficiency cannot be excluded without metabolite testing. Supplement use reported in the open inventory was far less common than deficiency for every nutrient: 168 participants (21.2%) reported a vitamin D product, 22 (2.8%) an iron product and 17 (2.1%) a vitamin B12 product (Table 2).

### 3.3. Biochemical Status by Supplement Use

**Figure 1:**
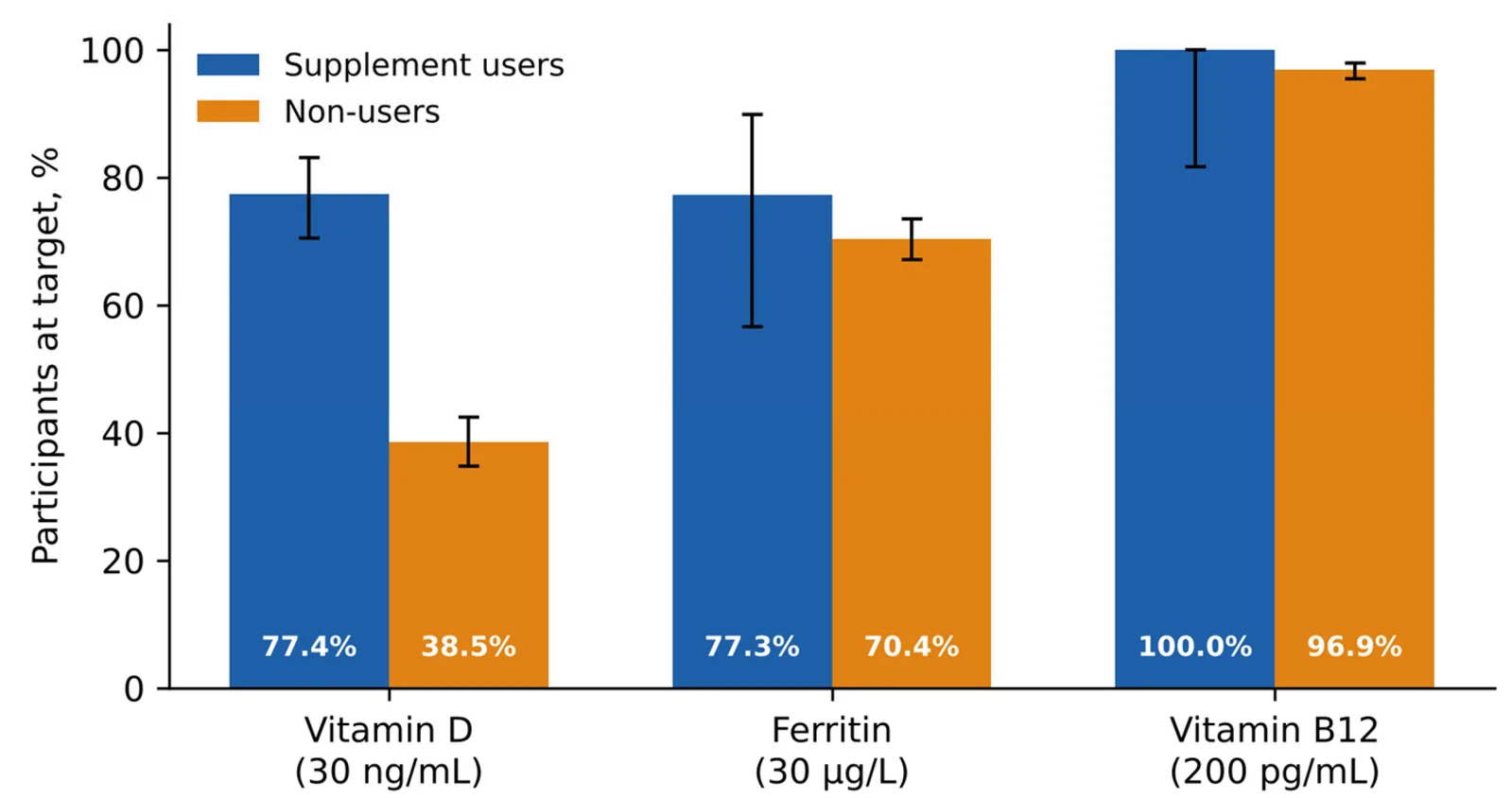
Proportion of participants at the biochemical target by supplement use reported in the open inventory, with Wilson 95% confidence intervals. Targets: 25-hydroxyvitamin D 30 ng/mL or more (168 users, 623 non-users); ferritin 30 µg/L or more (22 and 767); vitamin B12 200 pg/mL or more (17 and 774).

Vitamin D supplement users had better status than non-users: median 25(OH)D 37.0 ng/mL (IQR 30.9 to 49.7) versus 25.9 ng/mL (18.4 to 35.4), *p* < 0.001, and 130 of 168 users (77.4%, 95% CI 70.5 to 83.1) reached 30 ng/mL or more compared with 240 of 623 non-users (38.5%, 34.8 to 42.4; crude odds ratio 5.5, 3.7 to 8.1) (Figure 1, Table 3). Adjustment for age, sex, BMI, smoking and month of sampling changed the estimate little (odds ratio 5.5, 95% CI 3.7 to 8.3; adjusted mean difference in 25(OH)D 13.5 ng/mL, 10.9 to 16.0; adjusted ratio of geometric means 1.56, 1.43 to 1.69). The association was present in April (85/107, 79.4%, versus 125/322, 38.8%) and May (42/58, 72.4%, versus 110/278, 39.6%); the June stratum (3/3 versus 5/23) is too small to be informative (Table S2). At the 20 ng/mL threshold, 163 of 168 users (97.0%) and 441 of 623 non-users (70.8%) were at or above target. The distribution of 25(OH)D by supplement use is given in Table S4: 42 of 168 users (25.0%) and 48 of 623 non-users (7.7%) were at or above 50 ng/mL, and the two values above 100 ng/mL were both in users. Reclassifying prompted reports as use reduced the association substantially (Section 3.5). Among iron supplement users, 17 of 22 (77.3%, 56.6 to 89.9) had ferritin of 30 µg/L or more versus 540 of 767 non-users (70.4%, 67.1 to 73.5; Fisher’s exact *p* = 0.64; crude odds ratio 1.4, 0.5 to 3.9; adjusted 2.2, 0.8 to 6.1); median ferritin was lower in users (38.3 versus 50.2 µg/L, *p* = 0.14), consistent with supplementation being started in response to depletion. All 17 vitamin B12 users were above 200 pg/mL (100%, 81.6 to 100), compared with 750 of 774 non-users (96.9%, 95.4 to 97.9); attainment did not differ (Fisher’s exact *p* = 1.0), and the user groups for iron and vitamin B12 were too small to characterise an association.

### 3.4. Distribution of the Deficient Population by Prior Diagnosis and Supplement Use

Despite the association between supplement use and better status, few of the people who were deficient reported the corresponding supplement in the open inventory. Of the 421 participants with 25(OH)D below 30 ng/mL, 38 (9.0%, 95% CI 6.6 to 12.1) reported a vitamin D product, and of the 187 below 20 ng/mL, five (2.7%); of the 232 with iron deficiency, five (2.2%, 0.9 to 4.9) reported iron; and none of the 24 with low vitamin B12 reported a B12 product.

**Figure 2:**
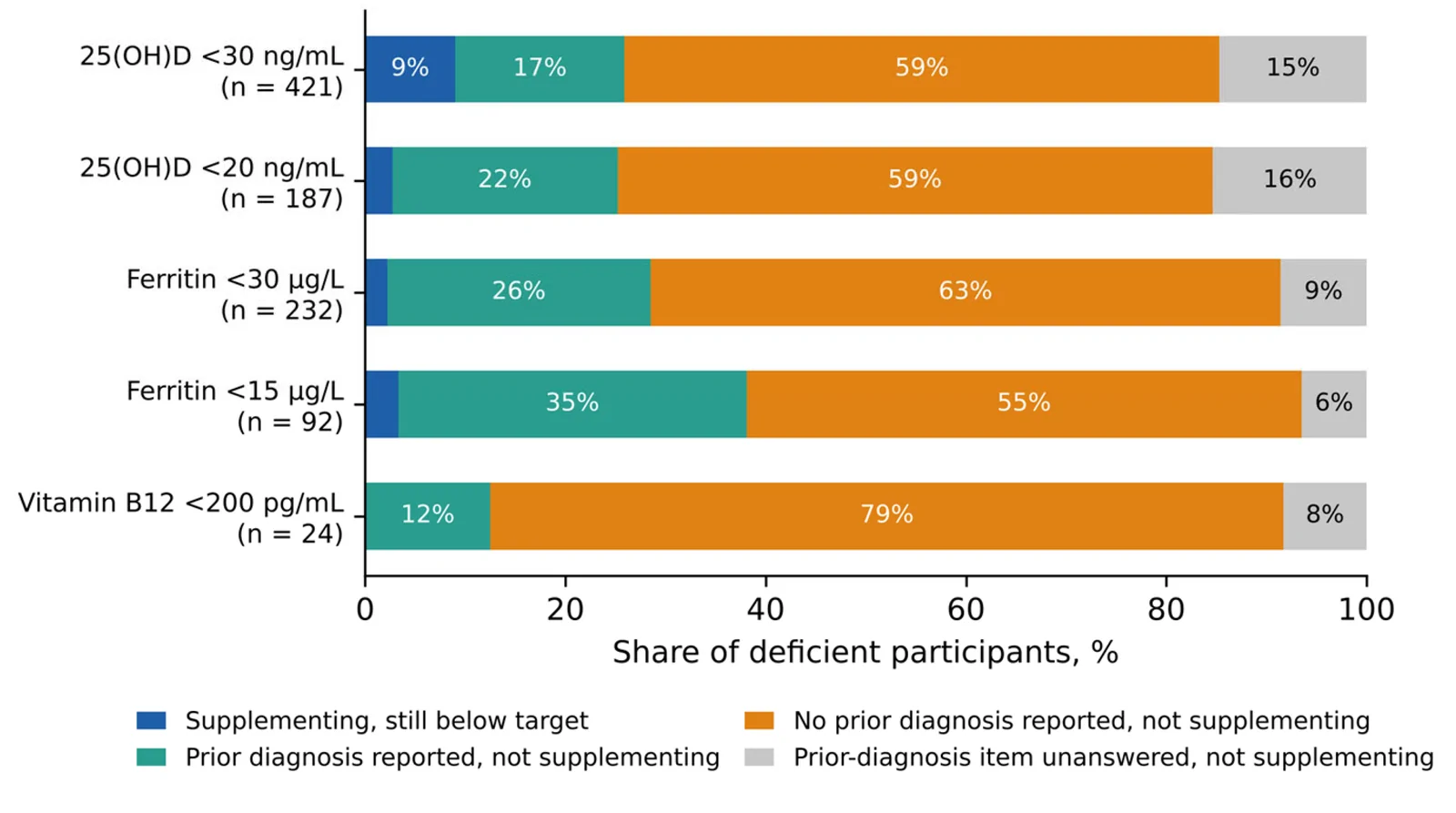
Composition of the deficient population for each nutrient and threshold, by reported prior diagnosis and supplement use (open inventory). Percentages are of all deficient participants; non-response to the prior-diagnosis item is shown separately. Among respondents, the group with neither diagnosis nor supplement is 70.2%, 70.3%, 69.2%, 59.3% and 86.4% (Table 4).

Partitioning the deficient population showed that the largest group in every case reported neither a previous diagnosis nor a supplement (Table 4, Figure 2). Among respondents to the prior-diagnosis item, this group comprised 250 of 356 for vitamin D (70.2%, 95% CI 65.3 to 74.7), 146 of 211 for iron (69.2%, 62.7 to 75.0) and 19 of 22 for vitamin B12 (86.4%, 66.7 to 95.3); counting non-respondents as undiagnosed gives 312 of 421 (74.1%), 166 of 232 (71.6%) and 21 of 24 (87.5%). A smaller group reported a previous diagnosis but no supplement (vitamin D 71, 16.9% of 421; iron 61, 26.3%; vitamin B12 3, 12.5%), and a small minority were supplementing yet still below target.

### 3.5. Sensitivity to the Instrument Used to Ascertain Supplement Use

**Figure 3:**
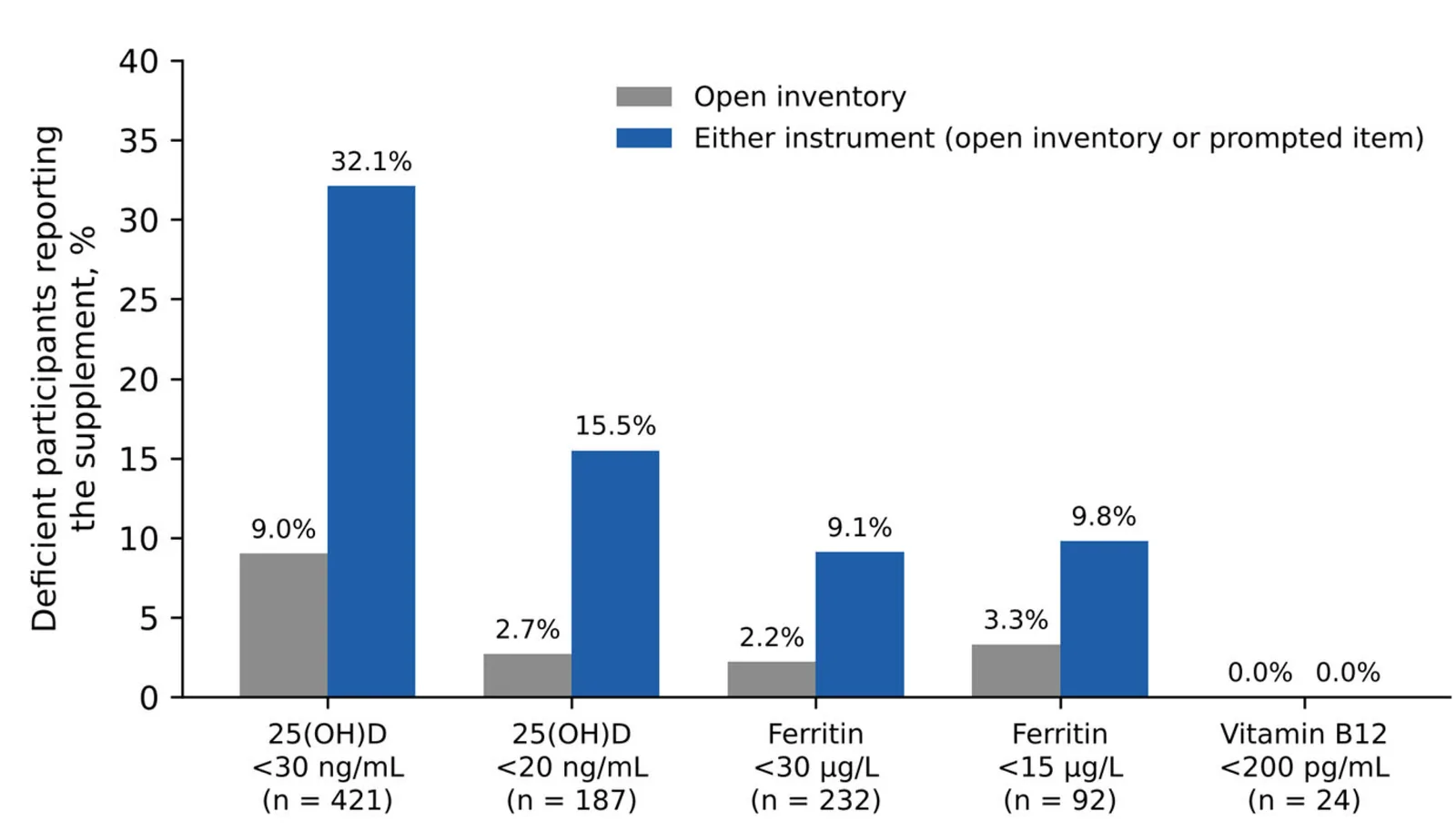
Proportion of deficient participants reporting the corresponding supplement in the open inventory and on either instrument, by nutrient and threshold (denominators 421, 187, 232, 92 and 24; Table 5).

The prompted vitamin D item, answered by 368 of the 421 participants with 25(OH)D below 30 ng/mL, elicited a report of vitamin D supplementation from 110 (29.9%), of whom only 13 had listed a vitamin D product in the open inventory. Combining the two instruments, 135 of 421 (32.1%) deficient participants reported vitamin D supplementation, against 38 (9.0%) in the open inventory alone, and the group with neither a reported diagnosis nor a reported supplement fell from 312 (74.1%) to 235 (55.8%). The open inventory captured two of 26 prompted reports among participants below 20 ng/mL, 11 of 84 between 20 and 30 ng/mL and 13 of 55 above 50 ng/mL. Above 50 ng/mL, 42 of 90 participants had listed a vitamin D product, and the prompted item added 42 more, so 84 of 90 (93.3%) reported use on either instrument; across the cohort, 307 of 791 (38.8%) reported vitamin D use on either instrument, a lower bound because the 280 participants between 30 and 49.9 ng/mL were never prompted. For iron, the prompted item, answered by 206 of 232 deficient participants, elicited 17 reports of iron supplementation, 16 of them absent from the open inventory; combining instruments gave 21 of 232 (9.1%) against five (2.2%), and the group with neither diagnosis nor supplement fell from 166 (71.6%) to 160 (69.0%). For vitamin B12, the prompted item reached only three of the 24 deficient participants and changed nothing (Table 5, Figure 3).

Because the prompted items were presented only on an abnormal result, every deficient participant who reported use on them was below target by construction, and the items cannot show how often supplementation fails to reach the target; the unconditional figure from the open inventory is that 38 of 168 vitamin D users (22.6%) were below 30 ng/mL. The prompted reports allow the effect of under-ascertainment on the comparison of users with non-users to be examined under the two scenarios of Section 2.5. In scenario A, reclassifying the 97 new reports below 30 ng/mL and the 42 above 50 ng/mL as use, with no additional users assumed in the never-prompted 30 to 49.9 ng/mL band, gave 172 of 307 users (56.0%, 95% CI 50.4 to 61.5) and 198 of 484 non-users (40.9%, 36.6 to 45.3) at target (crude odds ratio 1.8, 1.4 to 2.5; adjusted 1.8, 1.3 to 2.4; median difference 7.8 ng/mL, 32.9 versus 25.1; adjusted mean difference 11.8 ng/mL, 9.7 to 14.0). In scenario B, which additionally imputes 88 users in the 30 to 49.9 ng/mL band at the capture ratio observed above 50 ng/mL, 260 of 395 users (65.8%) and 110 of 396 non-users (27.8%) were at target (crude odds ratio 5.0, 3.7 to 6.8; adjusted 4.9, range 4.5 to 5.2 across replicates). The primary estimate of 5.5 and the scenario estimates of 1.8 and 4.9 are three values under three sets of assumptions about whom the open inventory missed; they do not bound the true association, which is also subject to confounding and reverse causation. The direction of the association was the same in all three. Stratifying by the timing of the interview, among the 401 participants interviewed on the day the result was reported, 71 of 86 users (82.6%) and 119 of 315 non-users (37.8%) were at target (adjusted odds ratio 7.8, 4.2 to 14.6), and among the 390 interviewed 1 to 25 days later, 59 of 82 users (72.0%) and 121 of 308 non-users (39.3%) (adjusted odds ratio 4.0, 2.3 to 6.9); among deficient participants, 15 of 211 (7.1%) interviewed on the day of the result and 23 of 210 (11.0%) interviewed later reported a vitamin D product in the inventory, and 52 of 191 (27.2%) and 58 of 177 (32.8%) on the prompted item.

### 3.6. Discrimination of Deficiency by Routine Characteristics

Routinely available characteristics discriminated deficiency poorly. For 25(OH)D below 30 ng/mL, the *C*-statistic was 0.59 (0.58 after optimism correction); deficiency was associated with younger age (odds ratio, OR 0.79 per decade, 95% CI 0.68 to 0.93) and higher BMI (OR 1.26 per 5 units, 1.07 to 1.48), with no association with sex (OR 0.98, 0.70 to 1.37). For 25(OH)D below 20 ng/mL, the *C*-statistic was 0.65 (0.64 corrected), with current smoking (OR 2.09, 1.37 to 3.20) and BMI (OR 1.41 per 5 units, 1.18 to 1.68) associated. For iron deficiency, the *C*-statistic was 0.72 (0.71 corrected), but discrimination rested almost entirely on female sex (OR 22.68, 9.15 to 56.21); age and BMI contributed little, and a model restricted to women reached a *C*-statistic of 0.58 (0.57 corrected) (Table 6).

### 3.7. Other Sensitivity Analyses

Applying the stricter thresholds did not change the pattern (Table 2, Table 4 and Table 5): at 20 ng/mL, 97.0% of vitamin D users and 70.8% of non-users were at target, and 70.3% of deficient respondents reported neither a prior diagnosis nor a supplement; at 15 µg/L, the corresponding share among respondents with iron deficiency was 59.3%. Counting the 22 participants who reported a B-complex product as vitamin B12 users did not change the finding that no participant with low vitamin B12 reported a B12-containing product because none of the 22 was deficient. Counting the eight participants who reported a multivitamin or multimineral product as exposed added one participant to the vitamin D-deficient supplementing group and one to the iron-deficient supplementing group. Counting non-respondents to the prior-diagnosis item as undiagnosed, the alternative basis, raised the estimate of the share with neither diagnosis nor supplement by one to five percentage points above the respondent-based estimate (Table 4). Of the 791 participants with a 25(OH)D result, 145 (18.3%) had a value between 27 and 33 ng/mL, within 10% of the 30 ng/mL threshold, which is about two coefficients of variation of the assay at that concentration; the corresponding number around 20 ng/mL (18 to 22 ng/mL) was 84, and around the ferritin threshold (27 to 33 µg/L) 53.

## 4. Discussion

In a screened corporate workforce, vitamin D supplement use was associated with better 25(OH)D status, yet most participants with biochemical deficiency of vitamin D, iron or vitamin B12 reported no supplement and no prior diagnosis. Vitamin D supplement users were twice as likely as non-users to meet the guideline target (77.4% versus 38.5%); their median 25(OH)D was 11 ng/mL higher; and adjustment for age, sex, BMI, smoking and month of sampling changed the estimate little. For iron and vitamin B12, the user groups (22 and 17 participants) were too small to characterise an association, and iron users had a lower median ferritin than non-users. At the same time, fewer than one in ten participants below 30 ng/mL, about one in fifty with iron deficiency and none of the 24 with low vitamin B12 listed the corresponding supplement in the open inventory, and even after a prompted question, the proportions were one in three and one in eleven. Roughly seven in ten deficient participants who answered the question reported neither a prior diagnosis nor a supplement.

The cross-sectional design limits what can be inferred from the first of these findings. Supplement users may have had higher 25(OH)D before they started; may differ in diet, sun exposure and health behaviour; or may have been prompted to supplement by an earlier result; in our data, users differed from non-users in physical activity and smoking but not in age, sex or BMI. Reverse causation operates in the opposite direction for iron, where supplementation typically follows documented depletion, and the lower median ferritin among iron users is the expected signature of that sequence [39,40,41]. The open inventory also under-ascertained use, and the two scenarios built on the prompted item show the consequence: reclassifying prompted reports as use gives an adjusted odds ratio of 1.8 if no user was missed among participants at 30 to 49.9 ng/mL, and 4.9 if the open inventory missed users there at the rate observed above 50 ng/mL, against 5.5 in the primary analysis; these are scenario estimates under stated assumptions, not limits on the true association. Because the interview followed the laboratory report by 0 to 25 days and asked about current use, some participants may have started a supplement after seeing a low result, or reported existing use more completely; the association was stronger among participants interviewed on the day of the result (adjusted odds ratio 7.8) than among those interviewed later (4.0), which is the pattern such result-dependent reporting would produce, although the proportion reporting use did not differ between the two groups. The association for vitamin D is therefore consistent with, but does not prove, an effect of supplementation; its direction is robust, its size is uncertain, and the crude difference of 11 ng/mL (8 ng/mL after reclassification) is of the size that dose–response trials report for daily doses in the 1000 to 2000 IU range [19], the doses most often named in the inventory. Among supplement users, there are also people in whom supplementation prevented a deficiency they would otherwise have had; the data cannot separate them from users who were replete regardless.

The second finding is less sensitive to these concerns because it does not rest on comparing users with non-users. Whatever the direction of causation, 70% of respondents with 25(OH)D below 30 ng/mL, 69% of those with ferritin below 30 µg/L and 86% of those with low vitamin B12 reported neither a diagnosis nor a supplement, and with the prompted item, the vitamin D figure was still 56% of all deficient participants. Supplement use in this cohort appears to have tracked health-consciousness rather than measured status, consistent with survey evidence that most users start supplements to improve general health rather than on medical advice [18]. Income and education were not recorded; because the screening was employer-funded and the products reported are inexpensive, awareness is a more plausible barrier than affordability in this setting, although the data cannot test it. The consequence is that a substantial, self-directed intervention accrued mainly to people who were not deficient at the time of testing, while most of the deficient were not reached. The prompted item adds a second observation: when asked directly, one in three participants below 30 ng/mL reported taking vitamin D. Because the item was presented only to participants below target, it cannot show how often supplementation fails to reach the target; the unconditional figure from the open inventory is that 22.6% of users were below 30 ng/mL, which may reflect doses, adherence or durations that were insufficient, a pattern described in clinic populations [22]; or supplementation started recently after an earlier low result.

Our prevalence estimates are lower than those of Polish surveys sampled in winter and spring. In 5775 adults of mean age 54.0 years examined in late winter and spring 2014, 89.9% had 25(OH)D below 30 ng/mL and 65.8% below 20 ng/mL [4]; in northern Poland, 98% of adults were below 30 ng/mL after winter and 85% after summer [5]. Our figures of 53.2% and 23.6% were obtained in April to June in a cohort 15 years younger, with 21% (open inventory) to at least 39% (either instrument) reporting vitamin D supplementation. They are consistent with later sampling and with the growth of supplement use since 2014; the 2023 national guidelines now recommend supplementation for the general adult population [34]. For iron, ferritin below 30 µg/L in 38.3% of women lies just below the 40 to 55% with ferritin at or below 30 µg/L reported for European women of reproductive age [8], and iron deficiency with anaemia in 4.6% of women (27 of 592) is in line with the 2 to 5% reported for iron-deficiency anaemia in that population [8]. For vitamin B12, 3.0% below 200 pg/mL is below the approximately 6% estimated for adults under 60 [11]; no national survey of vitamin B12 status in Polish working-age adults is available for comparison.

The practical question is how the majority who report neither a diagnosis nor a supplement could be reached. Our data argue against targeting on demographic grounds: 25(OH)D below 30 ng/mL was essentially unpredictable from age, sex, BMI and smoking (*C*-statistic 0.58), and although iron deficiency was better discriminated (*C*-statistic 0.71), this amounted to little more than “test women”, since within women, the *C*-statistic fell to 0.57. Variables that would plausibly improve prediction, such as dietary intake, menstrual blood loss, sun exposure and medication use, were not collected, so the models show the discrimination achievable with routinely available data; better prediction would require variables not collected here. Blanket supplement promotion is an alternative, and for vitamin D, the 2023 Polish guidelines already recommend it [34]; in this cohort, it would also be taken by the 38% of non-users who were already at target, and the upper end of the distribution carries a plausible risk of over-supplementation: a quarter of users (42 of 168) were at or above 50 ng/mL, against 7.7% of non-users, and the two values above 100 ng/mL were both in users [3,42]. A pragmatic combination would pair low-dose population advice with awareness of situations in which deficiency is likely (limited sun exposure, little outdoor activity, restricted intake of meat, fruit and vegetables, vegetarian or vegan diets, heavy menstrual bleeding, symptoms of anaemia) and with measurement where these apply. Measurement is the only route to the remainder, but whether adding 25(OH)D or ferritin to routine panels is worthwhile depends on yield, harm and cost that this study did not assess, and current Endocrine Society guidance advises against routine 25(OH)D testing in healthy adults under 75 [35]. Whether workplace micronutrient screening followed by targeted repletion improves status at acceptable cost is a question for prospective evaluation [23,24]; the present data quantify the unrecognised burden and do not evaluate screening.

These data bear on food fortification, which the Academic Editor asked us to consider. Poland requires fat spreads to be fortified with vitamins A and D, up to 7.5 µg of vitamin D per 100 g, and mandates no other vitamin D fortification [43]; wider fortification has not been customary in the country [44]. In Finland, systematic fortification of fluid milk products and fat spreads from 2003 raised mean 25(OH)D in a representative adult sample from 48 to 65 nmol/L (19 to 26 ng/mL) between 2000 and 2011, while supplement use rose from 11% to 41% [45]. A European guidance paper argues for systematic fortification on the ground that supplement use tracks healthier lifestyles and higher socioeconomic status and so does not reach the people at highest risk [46]; the present cohort, in which 70% of deficient respondents reported neither a diagnosis nor a supplement, while a quarter of users were above 50 ng/mL, shows that pattern in a working population. The case differs by nutrient. For vitamin D, with 53.2% below 30 ng/mL and 23.6% below 20 ng/mL in late spring, together with the poor reach of self-directed supplementation, fortification is the population-level option that deserves evaluation in Poland, with intake kept below the upper level of 100 µg per day [46]. For iron, depleted stores in 29.4% but iron-deficiency anaemia in 4.6% of women and depletion in 2.5% of men argue for identifying and treating women rather than for mass fortification, which would also reach the 97.5% of men who are replete. For vitamin B12, 3.0% below 200 pg/mL gives no case for fortification. The present data describe one workforce and cannot estimate the effect or safety of fortification in Poland; a national survey with standardised 25(OH)D measurement before and after any change in policy, as conducted in Finland [45], would be the appropriate evaluation.

Strengths and limitations. The principal strength is the ascertainment of supplement use from an open inventory administered to every participant without reference to laboratory results by the system, with explicit mapping rules; condition-specific follow-up questions, which are triggered by abnormal values, were excluded from the primary analysis because using them would have conditioned exposure on outcome, and they were used instead to examine under-ascertainment. Deficiency was defined by external guideline thresholds reported at two levels, prior diagnosis was asked of all deficient participants, measurements came from an accredited laboratory network on automated immunoassays, and several nutrients were examined in the same cohort.

Several limitations constrain interpretation. First, the design is cross-sectional, so the association between supplement use and status cannot be read as a causal effect; residual confounding by health behaviour is likely, and for iron, reverse causation is expected. Second, the open inventory under-ascertains supplement use: the prompted item identified 97 vitamin D users among deficient participants whom the inventory had missed, capture was lower among the deficient (8 to 13% of prompted reports) than among participants above 50 ng/mL (24%), and sensitivity scenarios that reclassify prompted reports as use gave adjusted odds ratios of 1.8 and 4.9 against 5.5 in the primary analysis; these are scenario estimates, not bounds, because they address under-ascertainment only. The proportion of the deficient who take nothing is an upper bound. Dose, formulation, duration and adherence were not analysed, and name-based mapping may misclassify multi-ingredient products. Third, ferritin was interpreted without a marker of inflammation in most participants; C-reactive protein was measured in 23. Values below 30 µg/L are specific for depleted stores, but deficiency masked by inflammation would be missed, and the interview items on inflammatory conditions, being presented only after a non-depleted ferritin, cannot measure it; counting every participant who reported such a condition and had ferritin of 30 to 69 µg/L as deficient gives 41.8%, and counting all participants with ferritin of 30 to 69 µg/L gives 65.5%, against 29.4% without masking; these are scenarios, not bounds, because the conditions were self-reported, and two of the three items were presented to a subset. The estimate is also sensitive to the threshold, falling to 11.7% at 15 µg/L. Fourth, vitamin B12 status rests on a single total serum concentration without methylmalonic acid, homocysteine or holotranscobalamin, and the 24 participants below 200 pg/mL are better described as having low serum vitamin B12 than as having functional deficiency; with so few cases, the observation that none reported a B12 product should not be generalised. Fifth, “deficiency” throughout is a biochemical classification on a single measurement; 145 participants had 25(OH)D within 10% of the 30 ng/mL threshold, about two coefficients of variation of the assay, and the two-threshold presentation is intended to show how much the estimates depend on the cut-off. Sixth, 25(OH)D was measured on two chemiluminescence assays whose allocation to individual samples was not recorded and which differ in imprecision near 30 ng/mL and in cross-reactivity with 24,25-dihydroxyvitamin D3; the imprecision figures are the manufacturers’, and a single threshold was applied to vitamin B12 and ferritin results from assays that are not harmonised across manufacturers; with 24 cases of low vitamin B12, a between-platform bias near 200 pg/mL would matter. Seventh, the prior-diagnosis item was unanswered by 15% of participants below 30 ng/mL and 9% of those with iron deficiency, prior diagnosis was self-reported, and “no reported prior diagnosis” is not the same as unawareness: recall failure, earlier testing without a diagnosis being communicated, dietary measures and discontinued supplementation all fall into this category; both estimates of the share are given. Eighth, the inventory asked about current use 0 to 25 days after the laboratory report, so participants may have seen their results before completing it and may have started a supplement in response; such result-dependent reporting or initiation would classify as users people whose measured concentration reflects status before supplementation and would bias the association towards the null, and the weaker association among participants interviewed after the day of the result (adjusted odds ratio 4.0 versus 7.8) is compatible with it. Reporting of use did not differ by timing (21.4% versus 21.0%), and more complete reporting among the deficient would have reduced the under-ascertainment we observed. Ninth, 25(OH)D was sampled in late spring, after the seasonal nadir, which lowers prevalence relative to winter surveys; users and non-users were sampled in the same months, users somewhat earlier, and the association held within April and May, so sampling time does not explain the difference between them. Finally, participants came from a single, predominantly office-based employer; 52.9% of those invited took part; and non-participants may differ in supplement use. The healthy-worker effect [47] and self-selection of the health-conscious would make the community burden, if anything, greater, whereas attendance by employees who suspected a problem would act in the other direction. Women made up 75.2% of the sample, all participants were aged 18 to 67 and only three were 65 or older, so the findings do not extend to adolescents or older adults; no pregnancy was reported among the 568 women asked, and income and education were not recorded.

## 5. Conclusions

In an ostensibly healthy workforce, vitamin D supplement use was associated with better 25(OH)D status after adjustment for age, sex, BMI, smoking and month of sampling, with the size of the association uncertain because the open inventory under-ascertained use; the iron and vitamin B12 data were too sparse to characterise an association, and iron users had lower median ferritin than non-users. Most biochemical deficiency of all three nutrients occurred in people who reported neither a prior diagnosis nor a supplement and who could not be identified from routine characteristics. Improving micronutrient status in such populations depends on finding the people who need supplementation and giving them an adequate dose, which in practice requires measurement.

## Figures and Tables

**Table 1:** Characteristics of the study population (N = 793).

Characteristic	All (N = 793)	Women (n = 596)	Men (n = 197)
Age, years, mean (SD)	39.4 (9.3)	39.3 (9.3)	39.6 (9.2)
Age range, years	18 to 67	18 to 65	21 to 67
BMI, kg/m^2^, mean (SD) ^a^	25.3 (4.8)	24.8 (5.0)	26.8 (3.7)
Underweight (below 18.5), n (%) ^a^	21 (2.7)	21 (3.6)	0
Normal weight (18.5 to 24.9), n (%) ^a^	404 (52.4)	332 (57.6)	72 (36.9)
Overweight (25 to 29.9), n (%) ^a^	241 (31.3)	150 (26.0)	91 (46.7)
Obesity (30 or more), n (%) ^a^	105 (13.6)	73 (12.7)	32 (16.4)
Current smoker, n (%) ^b^	127 (16.1)	95 (16.1)	32 (16.2)
Previously diagnosed comorbidities, median (IQR)	0 (0 to 1)	0 (0 to 1)	0 (0 to 1)
Any medicine or supplement reported, n (%)	383 (48.3)	294 (49.3)	89 (45.2)

^a^ Percentages are among the 771 participants with a valid BMI (576 women, 195 men). ^b^ Among the 787 participants with valid smoking data. SD, standard deviation; IQR, interquartile range; BMI, body-mass index.

**Table 2:** Prevalence of biochemical deficiency by threshold and sex, and supplement use reported in the open inventory.

Nutrient and Threshold	Tested, n	Deficient, n (%)	Women, n/N (%)	Men, n/N (%)	Taking Supplement, n (% of Cohort) ^a^
25(OH)D < 30 ng/mL (primary)	791	421 (53.2)	312/594 (52.5)	109/197 (55.3)	168 (21.2)
25(OH)D < 20 ng/mL	791	187 (23.6)	139/594 (23.4)	48/197 (24.4)	168 (21.2)
Ferritin < 30 µg/L (primary)	789	232 (29.4)	227/592 (38.3)	5/197 (2.5)	22 (2.8)
Ferritin < 15 µg/L	789	92 (11.7)	91/592 (15.4)	1/197 (0.5)	22 (2.8)
Vitamin B12 < 200 pg/mL	791	24 (3.0)	18/594 (3.0)	6/197 (3.0)	17 (2.1)

^a^ Supplement use is independent of the threshold and is repeated for the two threshold rows of each nutrient.

**Table 3:** Biochemical status by supplement use reported in the open inventory.

Nutrient	Users, n	Median (IQR) in Users	Median (IQR) in Non-Users	*p* ^a^	At Target, Users, n/N (%; 95% CI)	At Target, Non-Users, n/N (%; 95% CI)	Crude OR for Target (95% CI)	Adjusted OR for Target (95% CI) ^b^
Vitamin D, ng/mL	168	37.0 (30.9 to 49.7)	25.9 (18.4 to 35.4)	<0.001	130/168 (77.4; 70.5 to 83.1)	240/623 (38.5; 34.8 to 42.4)	5.5 (3.7 to 8.1)	5.5 (3.7 to 8.3); n = 763
Ferritin, µg/L	22	38.3 (31.0 to 50.6)	50.2 (25.6 to 94.3)	0.14	17/22 (77.3; 56.6 to 89.9)	540/767 (70.4; 67.1 to 73.5)	1.4 (0.5 to 3.9)	2.2 (0.8 to 6.1); n = 761
Vitamin B12, pg/mL	17	554 (366 to 628)	411 (326 to 511)	0.044	17/17 (100; 81.6 to 100)	750/774 (96.9; 95.4 to 97.9)	not estimable ^c^	not estimable ^c^

^a^ Mann–Whitney U test for concentrations. ^b^ Logistic regression adjusted for age, sex, BMI, current smoking and month of sampling, among participants with complete covariates; targets 25(OH)D 30 ng/mL or more and ferritin 30 µg/L or more. ^c^ No vitamin B12 user was below target; Fisher’s exact *p* = 1.0 for attainment. IQR, interquartile range; OR, odds ratio; CI, confidence interval.

**Table 4:** Composition of the deficient population by reported prior diagnosis and supplement use (open inventory).

Group	25(OH)D < 30 ng/mL (n = 421)	25(OH)D < 20 ng/mL (n = 187)	Ferritin < 30 µg/L (n = 232)	Ferritin < 15 µg/L (n = 92)	Vitamin B12 < 200 pg/mL (n = 24)
Supplementing, still below target, n (%)	38 (9.0)	5 (2.7)	5 (2.2)	3 (3.3)	0
Prior diagnosis reported, not supplementing, n (%)	71 (16.9)	42 (22.5)	61 (26.3)	32 (34.8)	3 (12.5)
No prior diagnosis reported, not supplementing, n (%)	250 (59.4)	111 (59.4)	146 (62.9)	51 (55.4)	19 (79.2)
Prior-diagnosis item not answered, not supplementing, n (%)	62 (14.7)	29 (15.5)	20 (8.6)	6 (6.5)	2 (8.3)
Respondents to the prior-diagnosis item, n ^a^	356	158	211	86	22
Neither prior diagnosis nor supplement, among respondents, n/N (%; 95% CI)	250/356 (70.2; 65.3 to 74.7)	111/158 (70.3; 62.7 to 76.8)	146/211 (69.2; 62.7 to 75.0)	51/86 (59.3; 48.7 to 69.1)	19/22 (86.4; 66.7 to 95.3)
Neither prior diagnosis nor supplement; non-respondents counted as undiagnosed, n/N (%)	312/421 (74.1)	140/187 (74.9)	166/232 (71.6)	57/92 (62.0)	21/24 (87.5)

Percentages in the first four rows are of the deficient population. ^a^ Respondents include supplementing participants who answered the item (vitamin D 35 of 38 at 30 ng/mL and 5 of 5 at 20 ng/mL; iron 4 of 5 at 30 µg/L and 3 of 3 at 15 µg/L); the 3 and 1 supplementing non-respondents are counted in the first row only.

**Table 5:** Supplement use among deficient participants by ascertainment instrument.

Nutrient and Threshold	Deficient, n	Prompted Item Answered, n	Open Inventory, n (%)	Prompted Item, n	Either Instrument, n (%)	Neither Diagnosis nor Supplement, Open Inventory, n (%) ^a^	Neither Diagnosis nor Supplement, Either Instrument, n (%) ^a^
25(OH)D < 30 ng/mL	421	368	38 (9.0)	110	135 (32.1)	312 (74.1)	235 (55.8)
25(OH)D < 20 ng/mL	187	168	5 (2.7)	26	29 (15.5)	140 (74.9)	121 (64.7)
Ferritin < 30 µg/L	232	206	5 (2.2)	17	21 (9.1)	166 (71.6)	160 (69.0)
Ferritin < 15 µg/L	92	71	3 (3.3)	7	9 (9.8)	57 (62.0)	55 (59.8)
Vitamin B12 < 200 pg/mL	24	3	0	0	0	21 (87.5)	21 (87.5)

^a^ Non-respondents to the prior-diagnosis item counted as undiagnosed. Participants to whom the prompted item was not presented or who left it unanswered are counted as not reporting on it (Table S5).

**Table 6:** Multivariable logistic regression for deficiency, with discrimination.

Deficiency	n	*C*-Statistic, Apparent	*C*-Statistic, Optimism-Corrected ^a^	Female, OR (95% CI)	Age, OR per Decade (95% CI)	BMI, OR per 5 Units (95% CI)	Current Smoking, OR (95% CI)
25(OH)D < 30 ng/mL	763	0.59	0.58	0.98 (0.70 to 1.37)	0.79 (0.68 to 0.93)	1.26 (1.07 to 1.48)	1.40 (0.93 to 2.09)
25(OH)D < 20 ng/mL	763	0.65	0.64	1.11 (0.75 to 1.65)	0.73 (0.60 to 0.88)	1.41 (1.18 to 1.68)	2.09 (1.37 to 3.20)
Ferritin < 30 µg/L	761	0.72	0.71	22.68 (9.15 to 56.21)	0.81 (0.68 to 0.98)	0.86 (0.72 to 1.02)	0.72 (0.44 to 1.16)
Ferritin < 30 µg/L, women only	566	0.58	0.57		0.80 (0.66 to 0.96)	0.85 (0.71 to 1.02)	0.74 (0.45 to 1.20)
Ferritin < 15 µg/L ^b^	761	0.70	0.68	23.95 (6.49 to 211.75)	0.73 (0.57 to 0.94)	1.00 (0.79 to 1.23)	0.94 (0.49 to 1.71)

^a^ Harrell’s bootstrap optimism correction, 1000 resamples. ^b^ Firth’s penalised-likelihood estimates with profile-likelihood intervals because only one man was below 15 µg/L (maximum-likelihood estimate for female sex 35.6, Wald interval 4.9 to 258.1); *C*-statistics from the maximum-likelihood model. OR, odds ratio; CI, confidence interval; BMI, body-mass index.

## Data Availability

The dataset analysed in this study is not publicly available because it comprises de-identified individual-level health records collected within a commercial workplace preventive-health programme and is subject to data-governance and privacy restrictions. De-identified data may be made available from the corresponding author on reasonable request, subject to a data-sharing agreement and the relevant ethics and data-governance approvals. The product-mapping list (Table S1) and the analysis code are available from the corresponding author.

## Supplementary Materials

The following supporting information can be downloaded at: https://www.mdpi.com/article/10.3390/nu18213611/s1, Table S1: Products reported in the open medication and supplement inventory that were attributed to vitamin D, iron or vitamin B12, or examined in sensitivity analyses; Table S2: 25-hydroxyvitamin D by month of sampling and supplement use (open inventory); Table S3: Reference intervals attached to the results, the platform they indicate, and the analytical equipment reported by the laboratory; Table S4: Distribution of 25-hydroxyvitamin D by vitamin D supplement use reported in the open inventory; Table S5: Presentation and response of the conditional interview items among deficient participants; Table S6: STROBE checklist for cross-sectional studies. Reference [25] is cited in the Supplementary Materials.
