# Supplementary material for: Vitamin D Supplement Use Is Associated with Better Status, Yet Most Biochemical Vitamin D, Iron and Vitamin B12 Deficiency Is Unrecognised and Unsupplemented: A Cross-Sectional Study of a Screened Corporate Workforce in Poland
Source: Nutrients. 2026 Oct 30;18(21):3611. doi: 10.3390/nu18213611 (PMC13765095; doi:10.3390/nu18213611)
Supplement: Supplementary file 1 [file nutrients-18-03611-s001.zip › nutrients-4600123-supplementary.pdf]

Supplementary Material

Vitamin D Supplement Use Is Associated with Better Status, yet Most Biochemical Vitamin D, Iron and Vitamin B12 Deficiency Is Unrecognised and Unsupplemented: A Cross-Sectional Study of a Screened Corporate Workforce in Poland. Swadźba D., Makuch S., Agrawal S.

Table S1. Products reported in the open medication and supplement inventory that were attributed to vitamin D, iron or vitamin B12, or examined in sensitivity analyses.

Product as reported (lower case)	Entries, n	Vitamin D	Iron	Vitamin B12	B-complex (sensitivity)	Multivitamin (sensitivity)
witamina d3	61	yes				
witamina d3 2000	19	yes				
witamina d3 + k2	11	yes				
d3 mono 2000 witamina d3	10	yes				
ibuvit d3 4000 iu	10	yes				
witamilki witamina d3	10	yes				
d3+k2 osteocard	6	yes				
biotter vitamina d3 forte	5	yes				
witamina adek	5	yes				
witamina d3 + k2 (mk7) witaminy ojca grzegorza	4	yes				
d3 active	3	yes				
devikap	3	yes				
dekristol	2	yes				
ibuvit d	2	yes				
ibuvit d3 1000 + k2 mk-7 omega 3	2	yes				
ibuvit d3 2000 iu	2	yes				
vigantoletten max	2	yes				
witamina d3 2000 iu w oleju z krokosza barwierskiego	2	yes				
apod3 max	1	yes				
chela calcium d3	1	yes				
d-vitum	1	yes				
d-vitum forte k2	1	yes				
d3 mono 1000 witamina d3	1	yes				
ibuvit d3 2000 + k2 mk-7 omega 3	1	yes				
juvit d3	1	yes				
vigantol	1	yes				
vitrum d3 forte	1	yes				
wapń z witaminą d3	1	yes				

Product as reported (lower case)	Entries, n	Vitamin D	Iron	Vitamin B12	B-complex (sensitivity)	Multivitamin (sensitivity)
witamina d3; -forte	1	yes				
żelazo kwas foliowy + wit. c	5		yes			
sorbifer durules	3		yes			
szelazo+ sr	3		yes			
żelazo z witaminą c	3		yes			
feroplex	2		yes			
tardyferon	2		yes			
tardyferon-fol	2		yes			
tarvit żelazo	2		yes			
sorbifer durules - (ir)	1		yes			
witamina b12	9			yes		
b12 forte bio-complex	3			yes		
b12-vitum	2			yes		
diab12	2			yes		
naturell witamina b12; -forte	1			yes		
witamina b complex	16				yes	
neurovit	2				yes	
witamina b-kompleks	2				yes	
laboratoria polfałódź b-complex	1				yes	
vitaminum b complex; -forte	1				yes	
femibion 0 planowanie ciąży	2					yes
multiwitamina	2					yes
bodymax plus	1					yes
centrum on	1					yes
mama dha; -premium	1					yes
pregna dha	1					yes
zdrovit witaminy i minerały	1					yes

Attribution rule: a product was attributed to a nutrient if the nutrient was named in the product name or the product was a single-nutrient branded preparation of that nutrient; combination products naming the nutrient were counted regardless of dose; attribution was by name and product composition was not consulted. Entries are counted per product: two participants listed two vitamin D products, one listed two iron products and one listed two multivitamin products, so the column sums (vitamin D 170, iron 23, multivitamin 9) exceed the user counts (168, 22, 8). Products listed only once are included. Prescription medicines and supplements not relevant to the three nutrients (for example omega-3, magnesium, vitamin C, levothyroxine) are omitted. B-complex and multivitamin products were not attributed in the primary analysis.

Table S2. 25-hydroxyvitamin D by month of sampling and supplement use (open inventory).

Month of sampling	Group	n	Median 25(OH)D, ng/mL	At or above 30 ng/mL, n (%)
April 2026	Supplement users	107	37.0	85 (79.4)
April 2026	Non-users	322	25.5	125 (38.8)

Month of sampling	Group	n	Median 25(OH)D, ng/mL	At or above 30 ng/mL, n (%)
May 2026	Supplement users	58	36.3	42 (72.4)
May 2026	Non-users	278	26.0	110 (39.6)
June 2026	Supplement users	3	43.1	3 (100)
June 2026	Non-users	23	26.8	5 (21.7)
All	Supplement users	168	37.0	130 (77.4)
All	Non-users	623	25.9	240 (38.5)

Month is that of the 25(OH)D result, reported within 4 days of the first result from the same sample (blood drawn 21 April to 16 June 2026). Users were sampled earlier than non-users (63.7% versus 51.7% in April; chi-squared p = 0.007). The June stratum (26 participants, 3 users) is too small to be informative. Logistic regression of attainment on supplement use adjusted for age, sex, BMI, current smoking and month (three categories): odds ratio 5.5 (95% CI 3.7 to 8.3), n = 763; linear regression: adjusted difference 13.5 ng/mL (10.9 to 16.0); log-scale model: ratio of geometric means 1.56 (1.43 to 1.69).

Table S3. Reference intervals attached to the results, the platform they indicate, and the analytical equipment reported by the laboratory.

Analyte	Reference interval on report	Results, n (%)	Platform indicated by the interval	Equipment and method reported by the laboratory
Vitamin B12	197 to 771 pg/mL	663 (83.8)	Roche cobas e, Elecsys Vitamin B12 II (electrochemiluminescence immunoassay)	Roche cobas e series (Pure, Pro, 8000, 6000), Abbott Alinity i, DiaSorin LIAISON XL; chemiluminescence immunoassay
Vitamin B12	187 to 883 pg/mL	44 (5.6)	Abbott Alinity i (chemiluminescent microparticle immunoassay)	
Vitamin B12	other intervals	84 (10.6)	third platform (DiaSorin LIAISON XL according to the equipment list)	
Thyrotropin	0.27 to 4.20 µIU/mL	678 (85.7)	Roche cobas e, Elecsys TSH	not requested
Thyrotropin	0.35 to 4.94 µIU/mL	105 (13.3)	Abbott Alinity i	
Thyrotropin	other intervals	8 (1.0)	other platform	
25(OH)D	30 to 50 ng/mL (laboratory target range, identical on all reports)	791 (100)	not identifiable from the report	DiaSorin LIAISON XL (LIAISON 25 OH Vitamin D TOTAL) and Abbott Alinity i (Alinity 25-OH Vitamin D); chemiluminescence immunoassay; allocation of samples between the two not recorded
Ferritin	30 to 200 µg/L (women), 30 to 400 µg/L (men) (laboratory reference intervals, identical on all reports)	789 (100)	not identifiable from the report	Roche cobas c series (Pure, Pro, 8000, 6000) and Integra 400, Abbott Alinity c; spectrophotometric immunoturbidimetric assay
Blood count	laboratory reference intervals	792 (100)	not applicable	Sysmex XN series (XN-1000, XN-550, XN-9100 lines) and

Analyte	Reference interval on report	Results, n (%)	Platform indicated by the interval	Equipment and method reported by the laboratory
				Mindray BC-6000 series; fluorescence flow cytometry

The platform of individual samples was not recorded in the data export. The fourth column is an inference from the reference intervals printed on the reports; the fifth column is the equipment list provided by Diagnostyka S.A. in October 2026 for the analytes studied, which applies across its laboratory network.

Table S4. Distribution of 25-hydroxyvitamin D by vitamin D supplement use reported in the open inventory.

25(OH)D band, ng/mL	Supplement users (n = 168), n (%)	Non-users (n = 623), n (%)	All (n = 791), n (%)
Below 20	5 (3.0)	182 (29.2)	187 (23.6)
20 to 29.9	33 (19.6)	201 (32.3)	234 (29.6)
30 to 49.9	88 (52.4)	192 (30.8)	280 (35.4)
50 to 99.9	40 (23.8)	48 (7.7)	88 (11.1)
100 or above	2 (1.2)	0	2 (0.3)

The two values above 100 ng/mL were 111.0 and 154.2 ng/mL. The prompted vitamin D item was triggered below 30 and above 50 ng/mL; participants between 30 and 49.9 ng/mL were never prompted.

Table S5. Presentation and response of the conditional interview items among deficient participants.

Item	25(OH)D <30 ng/mL (n = 421)	25(OH)D <20 ng/mL (n = 187)	Ferritin <30 μg/L (n = 232)	Ferritin <15 μg/L (n = 92)	Vitamin B12 <200 pg/mL (n = 24)
Prompted vitamin D supplement item: presented / answered / yes	378 / 368 / 110	170 / 168 / 26	145 / 142 / 50	58 / 57 / 16	15 / 15 / 1
Prompted iron supplement item: presented / answered / yes	147 / 145 / 15	67 / 67 / 7	209 / 206 / 17	71 / 71 / 7	7 / 7 / 0
Prompted vitamin B12 supplement item: presented / answered / yes	27 / 27 / 10	14 / 14 / 4	15 / 15 / 6	5 / 5 / 3	3 / 3 / 0
Prior diagnosis of vitamin D deficiency: presented / answered / yes	421 / 356 / 82	187 / 158 / 44	213 / 183 / 34	85 / 71 / 9	24 / 20 / 4
Prior diagnosis of iron deficiency: presented / answered / yes	413 / 372 / 50	181 / 164 / 22	223 / 193 / 52	84 / 75 / 24	23 / 20 / 0
Prior diagnosis of iron-deficiency anaemia: presented / answered / yes	370 / 339 / 11	165 / 152 / 7	182 / 159 / 13	71 / 62 / 11	23 / 21 / 1
Prior diagnosis of anaemia: presented / answered / yes	12 / 11 / 2	6 / 6 / 1	17 / 14 / 2	12 / 11 / 1	2 / 2 / 0
Under medical care for anaemia: presented / answered / yes	7 / 7 / 1	6 / 6 / 1	11 / 11 / 1	11 / 11 / 1	0 / 0 / 0
Any iron item: presented / answered / any yes	419 / 392 / 61	186 / 173 / 29	231 / 211 / 65	92 / 86 / 35	23 / 21 / 1
Prior diagnosis of vitamin B12 deficiency: presented / answered / yes	413 / 355 / 18	183 / 157 / 9	229 / 185 / 10	91 / 74 / 3	24 / 22 / 3

Item	25(OH)D <30 ng/mL (n = 421)	25(OH)D <20 ng/mL (n = 187)	Ferritin <30 μg/L (n = 232)	Ferritin <15 μg/L (n = 92)	Vitamin B12 <200 pg/mL (n = 24)
Prior diagnosis of vitamin B12-deficiency anaemia: presented / answered / yes	395 / 348 / 1	174 / 152 / 1	219 / 182 / 2	88 / 74 / 1	21 / 19 / 0
Any vitamin B12 item: presented / answered / any yes	413 / 375 / 19	183 / 166 / 10	229 / 196 / 12	91 / 78 / 4	24 / 22 / 3

The export records every item presented by the interview, answered or not. Across the whole cohort the prompted vitamin D item was presented to 439 participants (428 answered, 165 yes), the prompted iron item to 242 (238 answered, 23 yes) and the prompted vitamin B12 item to 52 (52 answered, 20 yes); the physical-activity item was presented to 277 (273 answered). The partition in Table 4 uses, for each nutrient, the items in the rows "Prior diagnosis of vitamin D deficiency", "Any iron item" and "Any vitamin B12 item".

Table S6. STROBE checklist for cross-sectional studies.

Item	No.	Recommendation	Location in manuscript
Title and abstract	1	Indicate the study design in the title or abstract; provide an informative and balanced summary	Title; Abstract
Background/rationale	2	Explain the scientific background and rationale	Introduction, paragraphs 1 to 4
Objectives	3	State specific objectives, including any prespecified hypotheses	Introduction, last paragraph
Study design	4	Present key elements of study design early in the paper	Section 2.1
Setting	5	Describe the setting, locations and relevant dates, including periods of recruitment, exposure, follow-up and data collection	Section 2.1
Participants	6	Give the eligibility criteria and the sources and methods of selection of participants	Section 2.1
Variables	7	Clearly define all outcomes, exposures, predictors, potential confounders and effect modifiers	Sections 2.2 to 2.4
Data sources/measurement	8	For each variable of interest, give sources of data and details of methods of assessment	Sections 2.2 to 2.4; Tables S3 and S5
Bias	9	Describe any efforts to address potential sources of bias	Sections 2.3 and 2.5; Section 3.5; Discussion, limitations
Study size	10	Explain how the study size was arrived at	Section 2.1 (all programme participants)
Quantitative variables	11	Explain how quantitative variables were handled in the analyses	Sections 2.2 and 2.5
Statistical methods	12	Describe all statistical methods, including those used to control for confounding, subgroups, missing data and sensitivity analyses	Section 2.5

Item	No.	Recommendation	Location in manuscript
Participants	13	Report numbers of individuals at each stage of the study	Section 2.1; Section 3.1; Table S5
Descriptive data	14	Give characteristics of study participants and information on exposures and potential confounders; indicate the number with missing data	Section 3.1; Table 1; Section 2.4
Outcome data	15	Report numbers of outcome events or summary measures	Sections 3.2 and 3.3; Tables 2 and 3
Main results	16	Give unadjusted and adjusted estimates and their precision; report category boundaries	Sections 3.3 to 3.6; Tables 3 to 6
Other analyses	17	Report other analyses done, including sensitivity analyses	Sections 3.5 and 3.7; Tables S2 and S4
Key results	18	Summarise key results with reference to study objectives	Discussion, paragraph 1
Limitations	19	Discuss limitations, taking into account sources of potential bias or imprecision	Discussion, limitations paragraph
Interpretation	20	Give a cautious overall interpretation of results	Discussion, paragraphs 2 to 5; Conclusions
Generalisability	21	Discuss the generalisability of the study results	Discussion, limitations paragraph
Funding	22	Give the source of funding and the role of the funders	Funding; Conflicts of Interest

Checklist based on von Elm et al. [25].
